# Influence of lauric acid on the susceptibility of chickens to an experimental *Campylobacter jejuni* colonisation

**DOI:** 10.1371/journal.pone.0204483

**Published:** 2018-09-27

**Authors:** Julia Hankel, Johanna Popp, Diana Meemken, Katrin Zeiger, Martin Beyerbach, Venja Taube, Günter Klein, Christian Visscher

**Affiliations:** 1 Institute for Animal Nutrition, University of Veterinary Medicine Hannover, Foundation, Hannover, Germany; 2 Institute for Food Quality and Food Safety, University of Veterinary Medicine Hannover, Foundation, Hannover, Germany; 3 Institute of Food Safety and Food Hygiene, Freie Universitaet Berlin, Berlin, Germany, Germany; 4 Institute for Biometry, Epidemiology and Information Processing, University of Veterinary Medicine Hannover, Foundation, Hannover, Germany; 5 BEST 3 Gefluegelernaehrung GmbH, Twistringen, Germany; USDA-ARS, UNITED STATES

## Abstract

Among the organic acids, lauric acid has shown a high level of *in vitro* activity against *Campylobacter jejuni*. The prevalence and intensity of *C*. *jejuni* excretion at slaughter often becomes lower with increasing age. In higher-aged broilers on organic farms which often use other breeds, in turn, the prevalence of *C*. *jejuni* is sometimes higher at slaughter. The question then arises as to whether a diet with higher lauric acid concentrations, the age alone or the genetic breed might have an effect in the spread and intensity of an experimental *C*. *jejuni* infection *in vivo*. Therefore, two complete diets with or without 2% lauric acid from palm kernel fatty acids were offered to 450 chickens (ten subgroups à 15 birds, repetitions: n = 3) of two broiler and two layer breeds (Ross 308, Hubbard JA 757, Lohmann Dual and Lohmann Brown-Classic). All breeds were reared for 42 days, Lohmann Brown-Classic also for about 98 days. Twenty-one days before dissection, three seeder birds per subgroup were orally infected with a 1 mL inoculum of *C*. *jejuni* (4.46±0.35 log_10_ CFU/mL). Qualitative detection of *C*. *jejuni* in cloacal swabs was performed at days 2, 4, 7, 14 after inoculation and at dissection in all birds. Quantitative detection was performed on excreta samples of seeder birds at days 2, 11 and 17 after experimental challenge and on caecal samples of all birds at dissection. Two days after experimental inoculation, *C*. *jejuni* prevalence was higher in control birds without lauric acid supplementation (48.9% vs. 39.6%; P = 0.0462). Depending on age, two days after inoculation the *C*. *jejuni* prevalence in young Lohmann Brown-Classic chickens was significantly lower (37.8% vs. 61.1%) whereas at dissection it was higher (99% vs. 67%). At day 2 after inoculation *C*. *jejuni* counts in the excreta of young Lohmann Brown-Classic were lower in comparison to those in old ones (log_10_ CFU/g: 3.30±2.68 vs. 5.24±1.56). Eleven (log_10_ CFU/g: 5.14±1.13 vs. 4.16±0.82) and 17 days after inoculatioin (log_10_ CFU/g: 3.77±2.02 vs. 1.72±1.87) it was the reverse situation. At dissection, the carriage of *C*. *jejuni* in caecal content was higher in younger than in older birds (log_10_ CFU/g: 8.57±0.46 vs. 6.66±1.43). An effect of genetic breed on *C*. *jejuni* prevalence was seen at dissection, this being lowest in Lohmann Dual chickens (91% vs. 98.9% in other breeds). At d 17 after challenge, *C*. *jejuni* counts in the excreta of young Lohmann Brown-Classic were lower in comparison to Ross 308 and Hubbard JA 757 (log_10_ CFU/g: 3.77±2.02 vs. 5.21±0.85 and 5.62±0.90). Lohmann Dual chickens showed an intermediary excretion, this being only significant lower compared to Hubbard JA 757 (log_10_ CFU/g: 4.31±0.89). In summary, the effect of lauric acid is limited to the initial phase after experimental inoculation. A higher age at infection seems to lead to a more rapid limitation of the infection. The excretion of *C*. *jejuni* appears to decrease more rapidly in layer breeds than in broiler lines after experimental inoculation.

## Introduction

Campylobacteriosis is one of the world's most important diarrhoeal diseases in humans [1]. It was estimated that in 2010, *Campylobacter* was responsible for 166 million diarrhoeal illnesses and 31,700 Guillain–Barré syndrome cases worldwide [2]. In Europe, in the overwhelming majority of cases Campylobacter-associated diarrhoeal diseases is caused by the consumption of contaminated poultry meat [3]. One approach to eliminating *C*. *jejuni* in the food chain is to prevent the colonisation of broiler chickens [4]. The extent of contamination shows a positive correlation between the counts of Campylobacter in the caecal content and the number of bacteria on the carcass and the pectoral muscle [5].

In *in vitro* studies, lauric acid was one of the most effective acids for inhibition (<0.5 mM for *C*. *jejuni* ssp. *jejuni* CCM 6214T) in the growth of *C*. *jejuni* [6]. Also, short chain fatty acids have been tested *in vitro*, butyrate being the most successful one [7]. The addition of butyrate as a coated formulation in an *in vivo* experiment failed to reduce caecal colonisation [7] as did diets supplemented with 1% (wt/wt) noncoated caproic, caprylic, or capric acid sodium salt [8]. Substituting 1% soybean-oil with 1% Lodestar^TM^ C8-10 (Loders Croklaan, Wormerveer, the Netherlands; 56% C10, 30% C8, 10% C12, <3% C6, and <3%) showed an effect on the number of *C*. *jejuni* bacteria required to colonise 50% of inoculated broilers. This was estimated to be 200 times higher in broilers fed with supplemented feed than in control broilers [9]. Studies regarding the effect of lauric acid rich fats *in vivo* are rare. Palm kernel oil and coconut oil belong to the group of lauric acid rich oils [10]. In the physical refining of palm kernel oil, palm kernel fatty acid distillates are obtained as a by-product and contain more than 38% lauric acid [11, 12].

Under common rearing conditions of broilers, *C*. *jejuni* is rarely found in birds less than three weeks of age [13]. In older bird, the relationship between birds`age and *C*. *jejuni* prevalence shows no uniform results. On the one side, *Campylobacter* counts were 0.5 and 0.7 log_10_ units higher on carcasses of 42-d-old birds as compared to counts on carcasses of broilers 49 and 56 d of age, respectively [14]. On the other side, there are research groups who showed that the prevalence increases with the age of the birds in the form of a quick, usually unnoticed spread throughout the flock [15].

In conventional poultry farming, economically effective production systems are established, using modern breeds for fattening with high feed intake and body weight gains [16]. It is known from meta-analytically collected data that increased growth has a significant correlation with reduced immune function [17]. For example, there was a large (standard difference in means ≈-0.8) and a significant (P<0.001) negative effect of selection for body mass on immune function [17]. In organic poultry farming, however, other breeds are used. These slow-growing breeds are described as having a lower mortality rate [18] and a more robust immune system [19]. Therefore, it is surprising at first glance that *Campylobacter* prevalence in organic poultry farming is very high [20], sometimes significantly higher than in conventional farming [21]. *Campylobacter* spp. were isolated from 100% of organic broiler flocks, from 36.7% of conventional broiler flocks and from 49.2% of extensive indoor broiler flocks [21]. There are hardly any statements about *C*. *jejuni* prevalence in laying hens. In one study, *Campylobacter* could be detected at a high prevalence in caeca (53.3%) [22]. Therefore, it is not clear whether the differences in *C*. *jejuni* prevalence between production methods (conventional or organic) and direction of use (poultry intended for meat or egg production) can be explained by a different risk of infection from the environment, differences in feeding, the age of birds or by different genetics.

Therefore, to our knowledge, there are no *in vivo* studies describing the fate of lauric acid from palm kernel fat in the gastrointestinal tract of broiler chickens. Furthermore, in a second approach, the course and intensity of an experimental *C*. *jejuni* challenge under the influence of a lauric acid rich palm kernel fatty acid distillate in poultry of different age at challenge and breeds was studied. The objective of this experimental *C*. *jejuni* infection approach was to investigate, (I) the fundamental effect of lauric acid supplementation in poultry of different breeds and directions of use, (II) the effect of age at time of experimental infections, (III) or rather the effect of breeds for different directions of use on *C*. *jejuni* spreading in small groups of birds.

## Materials and methods

Animal experiments were performed in accordance with the German rules and regulations and approved by the Ethics Committee of Lower Saxony for Care and Use of Laboratory Animals LAVES (Niedersaechsisches Landesamt fuer Verbraucherschutz und Lebensmittelsicherheit; reference: 33.19-42502-05-15A500).

### Animals and housing

The birds used in the experiment were supplied as day-old chicks directly from the hatcheries. The chicks of the genetics CobbSasso 175 (CS175; only genetic for experiment 1), Ross 308 (R308), and Hubbard JA 757 (Hb757) were obtained from BWE-Brueterei Weser Ems, Visbek, Germany. Male chicks of the Lohmann Brown-Classic (LBC-A42, LBC-A98; male; two age groups) and Lohmann Dual (LD) breeds were supplied by Lohmann Tierzucht GmbH, Cuxhaven, Germany.

The experiments were divided into two parts: In the first part (Experiment 1), chickens of the genetics CS175 were used for analysing the concentrations of lauric acid in different parts of the gastrointestinal tract at dissection. The birds were given either a conventional complete diet or a lauric acid rich diet. In total, 32 chickens were kept in two different groups after the pre-experimental period of two weeks up to the end of day 42 of life. The experiment was conducted once.

In the second part (Experiment 2), identical diets (without/with lauric acid supplement) were offered in birds of different breeds. For this second experiment chicks of the genetics R308, Hb757, LBC and LD were used. Therefore, effects on the spread of an experimentally induced *C*. *jejuni* infection were analysed depending on diet (without/with lauric acid supplement), age of the chickens (LBC-A42, LBC-A98) and on genetics (R308, Hb757, LBC, LD) during infection.

For Experiment 2 (Table 1), four different breeds (R308, Hb757, LD, LBC) were reared under the same conditions in three independent repetitions (r = 3). Each breed consisted of 30 broilers which were randomly assigned to two subpopulations (15 birds, respectively). Only male birds from laying hybrids (LD and LBC) were used for the experiments. Birds of the LBC breed were also integrated into the experimental phase at different ages (LBC-A42, LBC-A98). Five birds per repetition, kept in a separate pen in the same room were used as sentinels. Therefore, the trials were based on a total of 465 chickens, conducted in three repetitions with 155 birds each.

**Table 1:** Experimental design showing distribution of birds based on breed, age and diet.

	Breed									
Age 1*	R308		Hb757		LD		LBC-A42			
Age 2^†^									LBC-A98	
Diet	CON	LA	CON	LA	CON	LA	CON	LA	CON	LA
Group size (n)	15	15	15	15	15	15	15	15	15	15
Repetitions (r)	3	3	3	3	3	3	3	3	3	3
Total birds (N)^‡^	45	45	45	45	45	45	45	45	45	45

R308 = Ross 308; Hb757 = Hubbard JA 757; LD = Lohmann Dual; LBC-A42 = Lohmann-Brown-Classic, dissection after 42 days; LBC-A98 = Lohmann-Brown-Classic, dissection after 98 days; CON = Complete diet with control fat; LA = complete diet with palm kernel fatty acids rich in lauric acid.

*pre-experimental period of 14 days–age at dissection: ~42 days.

†pre-experimental period of 63 (repetition 1) or 70 days (repetitions 2+3).

‡for each repetition five birds were used as sentinels (without *C*. *jejuni* challenge, without direct contact to the other groups, being kept in a separate pen in the same room).

**Fig 1:**
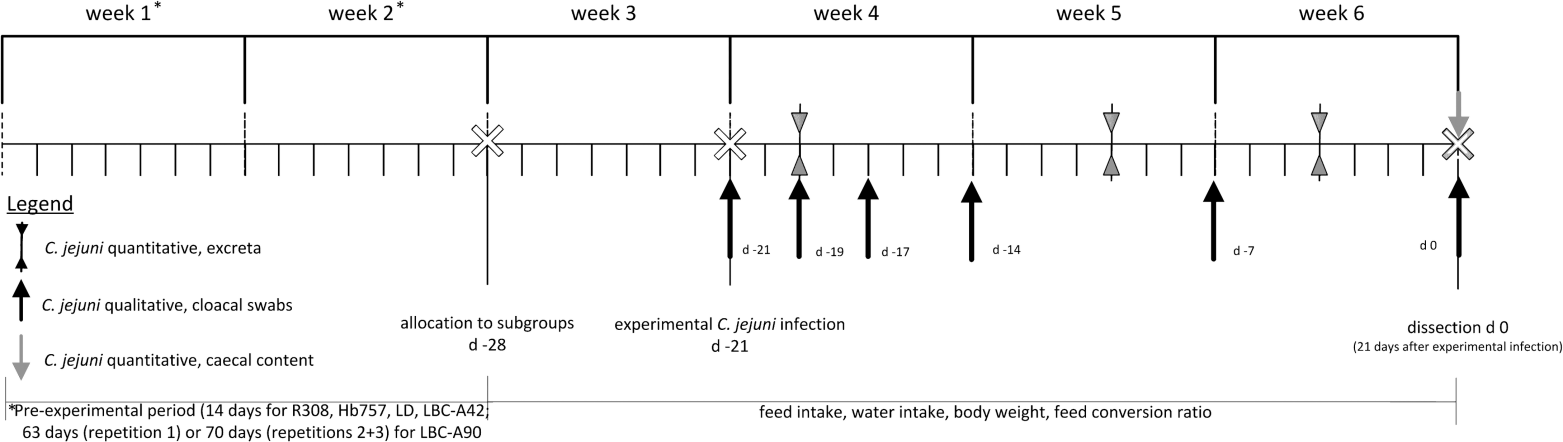
Schedule of the trial (Experiment 2) with actions, analyses and parameters in chickens of different breeds (R308 = Ross 308; Hb757 = Hubbard JA 757; LD = Lohmann Dual; LBC-A42 = Lohmann-Brown-Classic, dissection after 42 days; LBC-A98 = Lohmann-Brown-Classic, dissection after 98 days).

In the pre-experimental period (Fig 1), all birds were reared in identical floor pens littered with wood shavings. The pens were each equipped with an infrared lamp in order to reach a temperature at the outset of about 34–36°C. The temperature was lowered by about 1°C every two days, reaching a minimum temperature of about 20°C. The photoperiod beginning from d 4 was 16 h of light and 8 h of darkness during the whole trial.

After the fourteen-day rearing phase, all chickens in groups R308, Hb757, LD and LBC-A42 were transferred from the rearing to the infection unit of the animal house (security level 2). The experimental period started about four weeks before dissection (d-28). Birds in group LBC-A98 had been purchased seven (trial 1) or eight (trials 2+3) weeks prior to the start of the experimental period at a greater age. Therefore, these birds were transferred after an extended rearing phase to the infection unit at an age of about 63 or 70 days. At this time, birds of all genetic breeds and ages were randomly subdivided into ten subgroups for each repetition. Groups received either a control (CON) or the experimental lauric acid rich diet (LA; Table 2).

**Table 2:** Ingredient composition of the different experimental diets during the different fattening phases.

Item	Diet	Starter	Grower	Finisher	
	group			CON	LA
Diet composition (%)					
Wheat		42.8	52.2	49.3	49.3
Soybean meal		30.0	25.8	24.4	24.4
Corn		18.8	13.6	13.0	13.0
Fat/oil		3.78	4.75	2.91	2.91
Sunflower-, palm and soybean-oil		-	-	5.00	-
Palm kernel fat		-	-	-	5.00
Rape cake		-	-	2.58	2.58
CaCO_3_		1.65	1.33	0.95	0.95
Monocalcium phosphate		1.16	0.95	0.45	0.45
Sodium bicarbonate		0.17	0.18	0.19	0.19
Sodium chloride		0.17	0.13	0.11	0.11
Lysine		-	-	0.35	0.35
Methionine (hydroxy analogue)		0.37	0.31	0.27	0.27
Threonine		-	-	0.11	0.11
Narasine (mg/kg)		48.5	47.5	-	-
Nicarbacine (mg/kg)		48.5	47.5	-	-
Monensin sodium (mg/kg)		-	-	100	100
Further feed additives (difference to 100)		*	†	‡	‡

CON = Complete diet with control fat; LA = complete diet with palm kernel fatty acids rich in lauric acid.

*added to the diet: 11639 IU vitamin A, 4849 IU vitamin D3, 29.0 mg vitamin E, 14.5 mg Cu; 52.3 mg Fe, 107 mg manganese, 64.9 mg Zn, 1.90 mg iodine, 0.29 mg selenium, 485 IU 6-phytase; 1552 IU xylanases

† added to the diet: 11400 IU vitamin A, 4750 IU vitamin D3, 16.0 mg vitamin E, 14.2 mg Cu; 19.0 mg Fe, 66.5 mg manganese, 47.5 mg Zn, 1.90 mg iodine, 0.28 mg selenium, 475 IU 6-phytase; 1520 IU xylanases

‡ added to the diet: 12109 IU vitamin A, 5045 IU vitamin D3, 17.7 mg vitamin E, 15.1 mg Cu; 20.2 mg Fe, 70.6 mg manganese, 50.5 mg Zn, 2.02 mg iodine, 0.25 mg selenium, 454 IU 6-phytase; 1455 IU xylanases.

During the experimental period (d-28–d0) the birds were kept on solid floor pens in groups of 15 birds (“subgroup”) up to the end of the experimental period. A maximum stocking density of 30 kg per square metre applied to all groups. Due to the use of different breeds, metabolic body mass was applied as the standard dimension. A stocking density of 30 kg per square metre corresponds to a metabolic body mass of 23.3 kg^0.75^ per square metre. Due to the expected final body weight of the different breeds at the time of the dissection, the size of the boxes was then established. The base area of the feeding trough and the water lines, 0.17 square metres, was added to the calculated box size. As a result, the subgroups of different breeds had boxes with varying degrees of floor space available (R308: 1.53 m^2^, Hb757: 1.21 m^2^, LD: 1.04 m^2^, LBC-A42: 0.59 m^2^, LBC-A98: 1.17 m^2^ [Experiment 1] and 1.25 m^2^ [Experiment 2]). Movable partition walls made of stainless steel at a height of 60 cm made it possible to adapt the floor space to the desired stocking density. The boxes were separated from each other by 80 cm high completely closed plastic walls on the other three sides. The boxes were 106 cm wide but varied in depth. Between adjacent boxes there was also a distance of 106 cm. Boxes were littered with wood shavings (1 kg/m^2^).

### Feeding regime and diet

The diets used in the study were produced and delivered by a feed company producing compound diets (Best 3 Gefluegelernährung GmbH, Twistringen, Germany).

The rearing phase was divided into a one-week starter phase with a conventional pelleted starter diet (starter) and a subsequent seven-day phase with a commercially available pelleted grower diet (grower). All diets were offered *ad libitum*. During the first three days additional feed was available (about 300 g/d) on so-called Chickpaper to get the birds accustomed to the diet.

The different diets for the experimental period were designed in accordance with the recommendations for the energy and nutrient supply of the laying hens and fowls (broilers) of the Committee for Needs Standards of the Society for Nutritional Physiology [23]. The energy and nutrient content were comparable to the commercially available standard fattening diet (Table 3). The basic finisher feed for diets CON and LA was a common pellet diet which had been reduced by 2% in its fat content. In the CON groups, the diet was supplemented by 5% of a commercial standard fat (C 12 < 6%). In the LA groups, 5% fat enriched with palm kernel fatty acids including high levels of lauric acid (C 12 = 42–53%) was added as already described [24]. From the results of preliminary tests on the suitability of palm kernel fatty acids or combination of palm kernel fatty acids and capric acid and capric acid (data not shown), the use of a palm kernel product was preferred. The application amount was also determined in preliminary tests. Two different concentrations (2% and 5%) were tested. There was no negative effect on performance at the higher concentration. The higher concentration would also be the economically acceptable upper limit.

**Table 3:** Energy content and nutrient composition of the experimental diets during the different fattening phases.

Item	Diet	Starter	Grower	Finisher	
	group			CON	LA
Analysed nutrient composition (g/kg DM)					
ME (MJ/kg DM)		14.0	14.3	14.6	14.4
Crude ash		59.4	54.2	42.8	43.8
Crude fat		70.5	80.8	102*	91.5*
Crude fibre		21.1	22.4	26.9	26.5
Crude protein		247	238	217	220
Starch		416	431	441	435
Sugar		59.3	51.0	45.8	46.1
Ca		10.1	8.96	6.62	7.00
P		7.77	7.10	5.60	5.95
K		9.75	8.11	8.19	8.35
Na		1.41	1.48	0.98	0.99
Cl		1.84	1.60	1.23	1.24
Amino acids (g/kg DM)					
Arginine		16.2	15.2	14.1	15.3
Cysteine		4.50	3.96	5.49	4.24
Isoleucine		9.37	9.47	7.74	8.00
Leucine		22.2	18.1	16.1	16.7
Lysine		15.1	14.1	12.8	13.6
Methionine†		3.81	3.91	3.84	3.79
Phenylalanine		11.7	11.7	10.7	11.1
Threonine		10.2	9.16	8.69	9.33
Valine		10.1	10.8	9.77	10.0
Alanine		10.3	10.1	9.51	9.99
Aspartate		22.2	21.6	19.3	20.8
Glutamate		49.0	51.0	48.7	49.6
Glycine		9.63	9.49	9.33	9.58
Proline		15.8	17.0	14.6	13.8
Serine		12.4	11.7	11.4	12.0
Tyrosine		8.27	7.91	7.53	7.60
Fatty acids (g/kg DM)					
Caprylic acid		0.00	0.00	0.02	1.22
Capric acid		0.00	0.00	0.03	1.24
Lauric acid		0.69	1.04	0.50	21.0
Myristic Acid		0.32	0.45	0.44	7.64
Palmitic Acid		10.7	12.7	22.4	15.4
Stearic Acid		2.41	2.83	3.53	2.85
Oleic Acid		17.9	19.9	30.6	22.0
Linoleic Acid		31.5	36.2	40.0	27.5

CON = Complete diet with control fat; LA = complete diet with palm kernel fatty acids rich in lauric acid.

*By separate analysis of the control and test fat added to 5%, crude fat contents in the product of 955 g / kg of fresh substance and 812 g / kg of fresh substance were obtained. The free fatty acid content (C4:0 to C24:0) was almost identical (970 vs. 966 g). Therefore, the deviation of 10.5 g between CON and LA was, to a large extent (7.49 g), due to purely analytical causes. The diets are thus identical to the fat content. The calculation of the energy content is therefore also underestimated for the experimental diet (LA).

†only DL-methionine.

Circular feeding troughs were used (Crown Poultry Feeders, Nelson, New Zealand). In the rearing phase water was offered *ad libitum* in double-cylinder plastic bell drinkers. Later, drinking lines equipped with Top Nipples with a drinking cup were used (Big Dutchman International GmbH, Vechta-Calveslage, Germany). The water was treated with chlorine containing product (Virbac Clean Pipe, VIRBAC Tierarzneimittel GmbH, Bad Oldesloe, Germany) at a concentration of 0.3 mg free chlorine/L to kill any *C*. *jejuni* in the drinking water.

### Feed and chyme analysis

Diets were analysed by standard procedures in accordance with the official methods of the VDLUFA [25]. The analyses were always performed in duplicate. The dry matter content (DM) was determined by drying to the weight constancy at 103°C. The raw ash was analysed by means of incineration in the muffle furnace at 600°C for six hours. The total nitrogen content was determined by elemental analyser (Elementar, Hanau, Germany), which operates according to the principle of a catalytic tube combustion (DUMAS combustion method). Molecular nitrogen formed by reduction from nitric oxide was detected by a thermal conductivity detector. The nitrogen content was calculated with the device software. Therefore, the crude protein content of the sample was determined by multiplying with a constant factor of 6.25. The crude fat content was determined after acid digestion in the soxhlet apparatus. The content of crude fibre was determined after washing in dilute acids and alkalis. Starch determination was carried out polarimetrically (Polatronic E, Schmidt und Haensch GmbH & Co., Berlin, Germany). The sugar content was analysed in accordance with Luff-Schoorl method by titration with sodium thiosulphate. The mineral content was determined in accordance with the official methods [25] by atomic absorption spectrometry (Unicam Solaar 116, Thermo, Dreieich, Germany). Amino acids were determined by ion-exchange chromatography (AA analyser LC 3000, Biotronic, Maintal, Germany) and results evaluated in accordance with established methods [26]. Determining levels of medium and long chain fatty acids in the diet and intestinal content samples was carried out using established methods [27]. Exactly 200 mg of analytical material was placed in a glass tube. A methanol-hexane-tridecanoic-acid mixture was utilised as standard. Subsequently, acetyl chloride was added and the sample was heated, followed by the addition of potassium chloride solution. The measurement was carried out by gaschromatography (GC TRACE 1300, Thermo Scientific, Dreieich, Germany; SP-2560 Column, Supelco, Bellefonte, USA; carrier gas: nitrogen) after centrifugation with the superior hexane phase.

### Experimental design and sampling

#### Performance parameters

In Experiment 1, the individual body weight (BW) of birds was determined (PCE TB 30, PCE Instruments, Meschede, Germany) at the beginning and at the end of the feeding phase (d-28, d0) and the average daily feed intake (ADFI) on a subgroup basis to determine average daily weight gains (ADWG) and the feed conversion ratio (FCR).

In Experiment 2, the BW of the chickens was recorded individually at the start, at the time of experimental challenge and at the end of the experimental period (d-28, d-21 and d0). The ADFI was recorded at the level of the subgroup. Each subgroup had a 30 L polypropylene feed bucket into which the diet for the corresponding subgroup for the whole experimental period was stored. The feeding troughs were filled daily from these buckets. At the end of the trial, residuals in the troughs and buckets were weighed at a subgroup level (Systemwaage PCE TB 30, PCE Instruments, Meschede, Germany). The fresh drinking water for the storage tanks of the specific subgroups was weighed before filling the tanks daily.

#### Sampling–Experiment 1

The lauric acid content in the contents of the crop, the gizzard, anterior small intestine (ASI; end of muscular stomach to diverticulum vitellinum), posterior small intestine (PSI; diverticulum vitellinum to caeca) and caeca were determined exactly 42 days (d0) after hatch (Analysis described in the section "Feed and chime analysis").

#### Experimental challenge and sampling–Experiment 2

Prior to the described experimental challenge, *Campylobacter* exclusion diagnosis had been performed in two stages. First, three days before the experimental challenge five chickens per subgroup (n = 150/450) had been tested by means of a cloacal swab (Cary Blair smear test system, Suesse Labortechnik GmbH & Co. KG, Gudensberg, Germany) regarding a possible excretion of *C*. *jejuni*. If these birds had been positive, the experiment would not have been carried out. This was a termination criterion for the approved animal experiment. It takes about three days to confirm the Campylobacter-free status. Therefore, the first *C*. *jejuni* analysis had to be carried out three days before starting the experimental challenge. To justify the continuation of the experiments, all birds (n = 450/450) were again sampled prior to the experimental challenge (on the same day). If a bird had been positive at this stage, the experiment could have been stopped three days after the experimental challenge, when results of sampling would have been confirmed. This procedure has to taken place to ensure absolutely comparable starting conditions, i.e. Campylobacter-free status as a result of the second pre-sampling.

The experimental challenge with *C*. *jejuni* took place at d 21 in all subgroups. In each subgroup, three out of 15 broilers in a box were administered orally with a *C*. *jejuni* inoculum. When using pathogens with a low infection dose and a high tendency to spread and simultaneously keeping the birds in small groups, it is legally obliged to reduce the number of primary-infected birds to the lowest necessary level. This is preferable because the process of application of the pathogen can be seen as a burden. Therefore, only three out of 15 chickens were experimentally infected. In the case of natural infection in a flock it can also be assumed that the entry of the pathogen takes place at one location. Therefore, additionally the spread within the groups in case of partial infection is a particularly interesting parameter to test the efficiency of a dietetic concept as a model for the situation in practice. A direct comparison of the dietary concepts with respect to the quantitative excretion of Campylobacter was carried out exclusively by determining the number of Campylobacter in the excreta of primarily infected birds. A field strain of *C*. *jejuni* was used for the experimental challenge [28]. This isolate was identified as *C*. *jejuni* both culturally and mass-spectrometrically (MALDI-TOF MS) in a commercial laboratory (AniCon Labor GmbH, Hoeltinghausen, Germany). The advantage of this procedure is that only previously cultivated bacteria can be used for identification using MALDI-TOF. In our case, this means that we have also reliably identified the "infectious agent". Only one strain of Campylobacter was used for inoculation. This was done because muscle samples from the experiments should still be used for further questions [24]. Muscle samples from the experiment were spiked with a *Campylobacter coli* strain (*Campylobacter coli* strain DSM 4689). Lauric acid was present in higher concentrations in the muscle samples of birds offered the lauric acid supplement. In these *in vitro* experiments reduction of viable *Campylobacter coli's* was tested on the meat surface. In order to simplify the diagnosis of exclusion (possible contamination of the sample material with *Campylobacter jejuni*), only one pathogen was used in our inoculation model. The conserved strain for experimental inoculation was recultivated on solid selective culture media (mCCD agar; Oxoid Germany GmbH, Wesel, Germany). After incubation at 41.5°C in a microaerobic atmosphere for 43.5 hours, the challenge strain was used in its stationary growth phase (24 h to 48 h) for preparing the challenge inoculum. An isotonic 0.9% sodium chloride solution was used as the basis for the infection bouillon. A defined density of *C*. *jejuni* was adjusted (0.5 McFarland units corresponding to about 1 × 10^7^ CFU *C*. *jejuni* per mL) using a densitometer (DEN 1B, biosan SIA, Riga, Latvia). To obtain a challenge dose of about 10,000 CFU/1 mL of challenge inoculum, the inoculum was further diluted with sodium chloride. Three parallel batches were prepared. For the experimental challenge, three out of 15 birds were randomly selected. Infection bouillon was administered orally by means of a button cannula (single-button cannula, sterile, 1.0×100 mm, Meiser Medical GmbH, Neuenstein, Germany). Each of the three identically prepared challenge inocula was used for only one bird per subgroup.

In the experimental period, qualitative detection of *C*. *jejuni* took place on the day of experimental challenge (d-21), at further sampling points (d-19, d-17, d-14, d-7) and on the day of dissection (d0) in all birds (Fig 1). Quantitative detection of *C*. *jejuni* was done three times in seeder birds (three birds per subgroup) at d-19, d-10 and d-4. For excreta collection, the corresponding chickens were individually placed in purified, disinfected 10 L plastic buckets (ᴓ 26.5 cm) to collect fresh excreta. At dissection, samples of ceacal content were taken to analyse the counts of *C*. *jejuni* in this material.

In sentinel birds (five birds per repetitions, housed in a separate box in the security level 2-flock) regular spotchecks were carried out on the excretion of *C*. *jejuni*.

#### Dissection

Following the experimental period (d-28–d0), all chickens were dissected. Anaesthesia and killing of birds were carried out in accordance with EC 1099/2009. Anaesthesia was done by head stroke, after that the birds were bled. The contents of the different sections were removed under sterile conditions and placed in a screw cup (screw cup 100 mL, PP, Sarstedt AG & Co., Nuembrecht, Germany) for further analysis.

### Bacteriological analyses

The qualitative bacteriological examination was based on the DIN EN ISO 10272–1:2006, taken from the official collection of analysis methods in accordance with § 64 LFBG. First, pre-enrichment was performed in a liquid selective nutrient medium (Bolton Boullion). For this purpose, 500 mL of a basic nutrient medium (Bolton Brothbase, VWR International GmbH, Darmstadt, Germany) were first heated to 50°C in a water bath (GFL Gesellschaft fuer Labortechnik GmbH, Burgwedel, Germany). Lyophilised additives (Modified Bolton Broth Selective Supplement, Oxoid Deutschland GmbH, Wesel, Germany) were resuspended in a mixture of 2.5 mL ethanol and 2.5 mL autoclaved water. Afterwards, the additives were first transferred to the base nutrient medium, 25 mL lysed horse blood (Oxoid Deutschland GmbH, Wesel, Germany) subsequently being added.

The sample matrix to be examined was incubated in a one-to-nine ratio (sample:bolton-boullion) in sterile 5 mL tubes mounted with a vent cap (Sarstedt AG & Co., Nuembrecht, Germany). Incubation lasting 4 h at 37°C was followed by 44 h±4 h at 41.5°C in a microaerobic atmosphere (oxygen content of 5%±2%, carbon dioxide content of 10%±3%) in a CO_2_ incubator with O_2_ control (CB 160, BINDER GmbH, Tuttlingen, Germany). After enrichment, the samples were streaked onto two solid selective culture media (mCCD agar and Karmali agar; Oxoid Germany GmbH, Wesel, Germany) by sterile 10 μL inoculation loops. The incubation of the inoculated selective culture was carried out again for 44 h±4 h at 41.5°C in a microaerophilic atmosphere. Individual colonies were analysed to confirm the presence of Campylobacter. This was done by phase contrast microscopy (Distelkamp-Electronic, Kaiserslautern, Germany) and biochemical methods (apiCampy, bioMérieux SA, Marcy- l`Etoile, France).

For quantitative bacteriological examination a 10-fold dilution series (0.5 g sample material in 4.5 mL of sterile Phosphate Buffered Saline—PBS) was made with PBS (Phosphate Buffered Saline, Oxoid Germany GmbH, Wesel, Germany). In duplicate, 100 μL of each dilution was plated onto mCCD agar (Oxoid Germany GmbH, Wesel, Germany). After incubation in a microaerophilic atmosphere for 44 h±4 h at 41.5° C, the colonies were counted and an average value from the two duplicate experiments was taken for calculating the CFU/g intestinal content.

A diagnosis of exclusion concerning *Salmonella* spp. was performed as previously described [29]. Detection concerning coccidia was done weekly by an already described method [30].

### Statistical analyses

The statistical analyses were performed using the Statistical Analysis System for Windows, version 9.3, (SAS Institute Inc., Cary, North Carolina, USA). The group comparison for lauric acid concentrations in the gut contents was performed by a one-way analysis of variance (ANOVA) for independent samples on level of the different intestinal sections. Differences in concentration between the different sections of the gastrointestinal tract at group level were examined by means of one-way analyses of variance for repeated measures.

The performance data were analysed by means of analyses of variance for independent samples with regard to the influence factors “diet” at the level of the genetic breed x diet, “age” and “genetic breed”. The Ryan-Einot-Gabriel-Welsch multiple Range-Test was used for the multiple pairwise means comparisons. The distributions of the residuals from the linear models belonging to the analyses of variance were close to the normal one.

The qualitative target values concerning *C*. *jejuni* prevalence were assessed with Pearson’s Chi-square-Test for homogeneity and for low frequencies with Fisher’s exact test. For the statistical evaluation of counts of *C*. *jejuni* in excreta and caecal content samples, the data were logarithmised. The possible influence of the diet or of genetic breed on *C*. *jejuni* counts in the excreta of seeder birds was first determined by means of a one-way analysis of variance (ANOVA) for independent samples for the three different measurement times. Subsequently, the influence of time was determined by means of an analysis of variance for repeated measures. The counts of *C*. *jejuni* in the caecal content were analysed with respect to the factors “diet”, “age” and “genetic breed” by one-way analysis of variance (ANOVA) for independent samples. All statements of statistical significance are based upon p-values smaller than 0.05. This approach holds the comparisonwise error rate.

## Results

No mortality or animal health concern were recorded. Additionally, no samples were positive for *Salmonella* spp. or coccidia.

### Performance parameters

#### Experiment 1

At the end of the 42-day rearing period, the chickens showed a BW of 2150±321 g (CON) and 2035±313 g (LA), respectively (FCR–CON: 1.97, LA: 1.90).

#### Experiment 2

The BW and the ADWG at different time points as well as the FCR and the mortality showed no significant differences depending on diet within the specific genetic breed, with one exception among the older birds of the breed LBC-A98. Chickens which were offered a complete diet containing higher levels of lauric acid had a significantly lower weight gain in all analysed periods **(Table 4)**. Significant differences in BW as a function of age were present (LBC-A98>LBC-A42). The ADWG for LBC-A98 was significantly lower. The FCR was significantly higher for the older birds of the genetic breed LBC-A98 **(Table 5)**. Depending on the genetic breed, there were significant differences between all breeds depending on BW and ADWG at different time points in the following descending order: R308>Hb757>LD>LBC-A42. These differences existed at the beginning of the experimental period as well as at the end **(Table 5)**. The FCR was significantly the highest for the group R308, the lowest for LBC-A42 and intermediate for Hb757 and LD depending on the genetic breed.

**Table 4:** Growth performance, average weight gain, feed conversion ratio and mortality rate of poultry of different genetic breeds fed a diet without or with palm kernel fatty acids (n = 45 birds per breed and diet) in the last 28 days before dissection.

Item	Time	Diet																			
		R308				Hb757				LD				LBC-A42				LBC-A98			
		CON		LA		CON		LA		CON		LA		CON		LA		CON		LA	
		Mean	SD	Mean	SD	Mean	SD	Mean	SD	Mean	SD	Mean	SD	Mean	SD	Mean	SD	Mean	SD	Mean	SD
Body weight	d-28	451	47.2	444	54.4	310	41.5	317	31.5	212	32.2	213	26.9	133	14.6	132	14.3	1257	107	1276	136
	d-21	845	115	848	98.0	564	77.3	577	73.5	346	55.0	344	44.6	209	19.8	207	21.0	1397	110	1393	139
	d 0	2877	499	2794	328	1821	289	1897	303	1052	165	1050	133	601	60.6	589	57.6	1856	127	1811	144
ADWG (g/day)	d-28–d-21	56.3	11.0	57.6	9.15	36.2	6.10	37.1	6.51	19.2	4.48	18.7	3.49	11.0	1.26	10.8	1.35	20.0^a^	4.57	16.7^b^	6.99
	d-28–d 0	86.6	16.6	83.9	10.5	53.9	9.39	56.4	9.94	30.0	5.19	29.9	4.19	16.7	1.80	16.3	1.77	21.3^a^	2.41	19.1^b^	3.08
	d-21–d 0	96.8	19.0	92.7	11.8	59.9	11.0	62.9	11.4	33.7	5.60	33.6	4.58	18.6	2.15	18.2	2.14	21.8^a^	2.35	19.9^b^	3.75
FCR (kg/kg	d-28–d 0	1.54	0.01	1.57	0.08	1.77	0.14	1.69	0.11	1.82	0.07	1.84	0.04	2.15	0.01	2.09	0.09	3.60	0.25	3.74	0.35
Mortality (n)	d-28–d 0	No losses																			

R308 = Ross 308; Hb757 = Hubbard JA 757; LD = Lohmann Dual; LBC-A42 = Lohmann-Brown-Classic, dissection after 42 days; LBC-A98 = Lohmann-Brown-Classic, dissection after 98 days; CON = Complete diet with control fat; LA = complete diet with palm kernel fatty acids rich in lauric acid.

Lowercase letters (^a,b^) describe differences between performance parameters depending on diet within one genetic breed at *P*<0.05.

**Table 5:** Growth performance, average weight gain and feed conversion ratio of poultry of different genetic breeds (n = 90 per breed) and different ages (n = 90 per age group) in the last 28 days before dissection.

Item	Time	Age				Genetic breed							
		LBC-A42		LBC-A98		R308		Hb757		LD		LBC-A42	
		Mean	SD	Mean	SD	Mean	SD	Mean	SD	Mean	SD	Mean	SD
Body weight	d-28	132^b^	14.4	1267^a^	122	448^a^	50.8	314^b^	36.8	212^c^	29.5	132^d^	14.4
	d-21	208^b^	20.3	1395^a^	124	846^a^	106	570^b^	75.3	345^c^	49.8	208^d^	20.3
	d 0	595^b^	59.0	1834^a^	137	2836^a^	422	1859^b^	297	1051^c^	149	595^d^	59.0
ADWG (g/day)	d-28–d-21	10.9^b^	1.30	18.3^a^	6.10	57.0^a^	10.1	36.7^b^	6.29	18.9^c^	4.00	10.9^d^	1.30
	d-28–d 0	16.5^b^	1.78	20.2^a^	2.98	85.3^a^	13.9	55.2^b^	9.70	30.0^c^	4.69	16.5^d^	1.78
	d-21–d 0	18.4^b^	2.14	20.9^a^	3.26	94.7^a^	15.9	61.4^b^	11.3	33.6^c^	5.09	18.4^d^	2.14
FCR (kg/kg)	d-28–d 0	2.12^b^	0.07	3.67^a^	0.28	1.55^c^	0.05	1.73^b^	0.12	1.83^b^	0.05	2.12^a^	0.07

R308 = Ross 308; Hb757 = Hubbard JA 757; LD = Lohmann Dual; LBC-A42 = Lohmann-Brown-Classic, dissection after 42 days; LBC-A98 = Lohmann-Brown-Classic, dissection after 98 days. Lowercase letters (^a,b,c,d^) describe differences between performance parameters depending on diet within one genetic breed at *P*<0.05.

### Lauric acid concentration in the gastrointestinal tract

**Fig 2:**
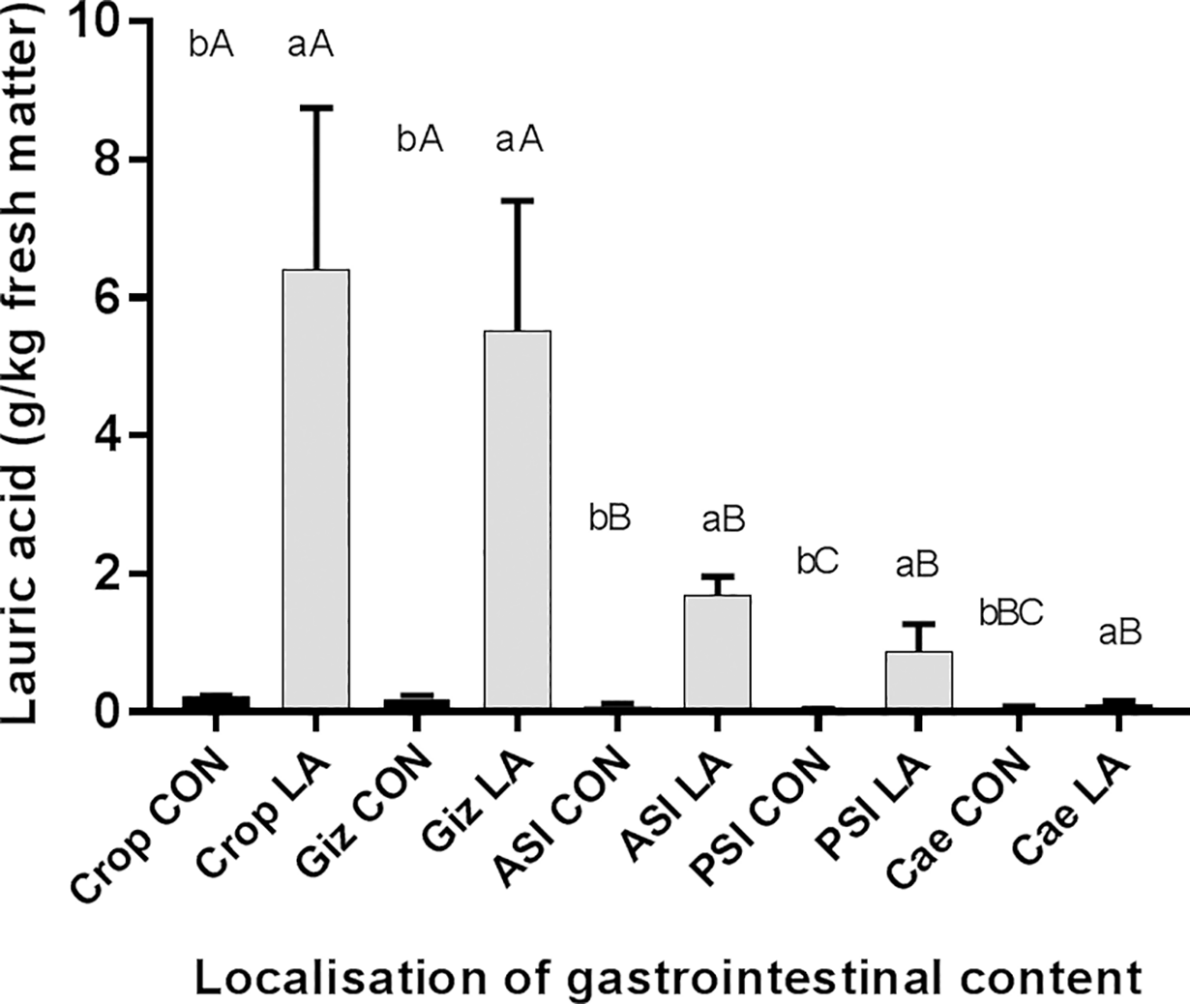
Concentration of lauric acid in different sections of the gastrointestinal tract of broiler chickens (n = 5/group; genetic breed: CobbSasso 175; Crop = content of the crop; Giz = content of the gizzard; ASI = content of the anterior small intestine; PSI = content of the posterior small intestine; Cae = content of the caecum; lowercase letters(^a,b^) describe differences between concentrations in lauric acid at one location at *P*<0.05; uppercase letters (^A,B^) describe differences between concentrations in lauric acid between locations depending on different diets at *P*<0.05.

In Experiment 1, the uptake of the diet containing lauric acid in relation to the control diet resulted in significantly higher lauric acid concentrations in the content of the respective section in broilers of breed CS175 (Fig 2).

The concentrations were significantly higher in the crop (CON: 0.22±0.01; LA: 6.41±2.34 g/kg fresh matter) and in the gizzard (CON: 0.19±0.05; LA: 5.53±1.91 g/kg fresh matter), anterior small intestine (ASI; CON: 0.07±0.04, LA: 1.68±0.26 g/kg fresh matter), posterior small intestine (PSI; CON: 0.03±0.01, LA: 0.88±0.39 g/kg fresh matter) and caecum (CON: 0.06±0.04; LA: 0.11±0.04 g/kg fresh matter).

### Campylobacter excretion

The inocula for the experimental challenge contained an average of 4.46±0.35 log_10_ CFU *C*. *jejuni* per challenge dose (1 mL).

Before and at the time of the experimental challenge (d-21), all chickens in the experiment were *C*. *jejuni* negative in cloacal swabs (N = 450). Sentinel birds (N = 15) remained negative during the whole trial. Seeder birds in the 30 experimental subgroups were inoculated at d 21 before dissection. Already two days after this challenge, an excretion of *C*. *jejuni* could be seen (Table 6). At this day (d-19), the number of chickens with a positive result concerning cloacal sampling was significantly higher in the control group receiving the CON diet (CON: 110/225 *C*. *jejuni* positive; LA: 89/225 *C*. *jejuni* positive; P = 0.0462).

**Table 6:** Prevalence of *Campylobacter jejuni* positive cloacal swabs depending on diet, age and genetic breeds after experimental challenge in poultry 21 days (-21 to 0) before dissection.

Time	Diet*		Age^†^		Genetic breed^‡^			
	CON	LA	LBC-A42	LBC-A98	R308	Hb757	LD	LBC-A42
	n-pos							
-21	0	0	0	0	0	0	0	0
-19	110^a^	89^b^	34^b^	55^a^	33	43	34	34
-17	214	216	86	90	85	83	86	86
-14	225	224	90	90	90	90	90	90
-7	224	225	90	89	90	90	90	90
0	198	211	89^a^	60^b^	89^a^	89^a^	82^b^	89^a^

R308 = Ross 308; Hb757 = Hubbard JA 757; LD = Lohmann Dual; LBC-A42 = Lohmann-Brown-Classic, dissection after 42 days; LBC-A98 = Lohmann-Brown-Classic, dissection after 98 days; CON = Complete diet with control fat; LA = complete diet with palm kernel fatty acids rich in lauric acid.

*225 birds per diet

^†^90 birds per age group

^‡^90 birds per breed.

Lowercase letters (^a,b^) describe differences between values in a row depending on diet, age or rather between genetic breeds at *P*<0.05.

The number of *C*. *jejuni* positive cloacal swab samples was significantly different depending on the age of the birds (LBC-A42, LBC-A98) 19 days before dissection. The prevalence was significantly higher in older birds (LBC-A42: 34/90 *C*. *jejuni* positive; LBC-A98: 55/90 *C*. *jejuni* positive; P = 0.0017). On the day of dissection, the results were contrary because 30 out of 90 birds of LBC-A98 were *C*. *jejuni* negative, whereas only one bird of LBC-A42 showed a negative test result in the cloacal swab (P<0.0001).

At the end of the experiment (dissection: d0), significant differences were found depending on genetics. Out of a total of 360 birds, 11 cloacal swabs were *C*. *jejuni* negative in direct detection. Of these, 72.7% swabs belonged to chickens of genetics LD (P = 0.0059, Table 6).

The number of colony-forming units of *C*. *jejuni* in the excreta samples of experimentally infected chickens (seeder birds) did not differ depending on the feeding regime (CON, LA; Table 7).

**Table 7:** Quantitative counts of *C*. *jejuni* in excreta samples [log_10_ CFU/g] of experimentally infected birds depending on diet and age.

Time	Diet*				Age†			
	CON		LA		LBC-A42		LBC-A98	
	Mean	SD	Mean	SD	Mean	SD	Mean	SD
-19	4.07	2.31	4.54	2.20	3.30^bB^	2.68	5.24^aA^	1.56
-10	4.79	1.07	5.19	0.88	5.14^aA^	1.13	4.16^bB^	0.82
-4	3.92	1.97	4.33	1.93	3.77^aB^	2.02	1.72^bC^	1.87

CON = Complete diet with control fat; LA = complete diet with palm kernel fatty acids rich in lauric acid; LBC-A42 = Lohmann-Brown-Classic, dissection after 42 days; LBC-A98 = Lohmann-Brown-Classic, dissection after 98 days.

*45 birds (seeder birds) per diet allocated to 15 groups

^†^18 birds (seeder birds) per age level allocated to 6 groups.

Lowercase letters (^a,b^) describe differences between breeds depending on diet and age at *P*<0.05; uppercase letters (^A,B,C^) describe differences between sampling points within age groups (LBC-A42, LBC-A98) at *P*<0.05.

Two days after successful experimental challenge (d-19), the excretion of *C*. *jejuni* with the excreta in older birds was significantly higher (LBC-A42: 3.30±2.68 log_10_ CFU/g; LBC-A98: 5.24±1.56 log_10_ CFU/g). At the tenth (d-10) and fourth day (d-4) before dissection, however, the excretion of the older birds was significantly lower. In particular, immediately before dissection the seeder birds of LBC-A98 showed significantly lower numbers of *C*. *jejuni* in the excreta (LBC-A42: 3.77±2.02 log_10_ CFU/g; LBC-A98: 1.72±1.87 log_10_ CFU/g), resulting in differences of Δlog_10_ CFU/g = 2.05. The quantitative excretion over time in comparison to the two age groups reached a maximum in excretion at second sampling (d-10) in the younger birds (LBC-A42). In older birds of the same genetic breed (LBC-A98), however, excretion reduced significantly over time (Table 7).

The excretion of *C*. *jejuni* with the excreta differed only just before the dissection (d-4) between the genetic breeds (Table 8).

**Table 8:** Quantitative counts of *C*. *jejuni* in excreta samples [log_10_ CFU/g] of experimentally infected birds depending on genetic breed.

Time	Genetic*							
	R308		Hb757		LD		LBC-A42	
	Mean	SD	Mean	SD	Mean	SD	Mean	SD
-19	4.24^B^	2.52	4.19^B^	2.16	4.56	1.97	3.30^B^	2.68
-10	5.41^A^	0.92	5.42^A^	0.72	4.82	0.82	5.14^A^	1.13
-4	5.21^abAB^	0.85	5.62^aA^	0.90	4.31^bc^	0.89	3.77^cB^	2.02

R308 = Ross 308; Hb757 = Hubbard JA 757; LD = Lohmann Dual; LBC-A42 = Lohmann-Brown-Classic, dissection after 42 days.

*18 birds per genetic breed (seeder birds) allocated to 6 groups.

Lowercase letters (^a,b,c^) describe differences between breeds depending on time at *P*<0.05

uppercase letters (^A,B^) describe differences between times within genetic breeds at *P*<0.05.

The experimentally infected birds of the Hb757 breed showed a nearly two log step (Hb757: 5.62±0.90 log_10_ CFU/g; LBC-A42: 3.77±2.02 log_10_ CFU/g) higher excretion than birds of the LBC-A42 breed. Within the respective breeds, there were differences depending on the time of the sampling after experimental challenge. In birds off the R308 and Hb757 breeds, the excretion was less pronounced shortly after experimental challenge (d-19), whereas in birds of the laying breed (LBC-A42) the excretion was significantly the highest at the mean sampling point (d-10).

There was no significant effect of diet on the number of *C*. *jejuni* in the caecal content, even when comparing the feeding concepts within the genetic breeds (Table 9). Nonetheless, the Campylobacter counts in the caecum differed significantly depending on the age of the chickens (P<0.0001). The LBC-A98 chickens had significantly lower numbers of *C*. *jejuni* in the caecum (6.66±1.43 log_10_ CFU/g). The difference to LBC-A42 amounted to 1.91 log_10_ CFU/g so that these groups differed by almost 2 log steps.

**Table 9:** Quantitative counts of *C*. *jejuni* in caecal content samples [log_10_ CFU/g] of experimentally infected birds depending on diet and age.

Time	Diet*				Age^†^			
	CON		LA		LBC-A42		LBC-A98	
	Mean	SD	Mean	SD	Mean	SD	Mean	SD
Dissection	8.07	1.18	8.13	1.03	8.57^a^	0.46	6.66^b^	1.43

CON = Complete diet with control fat; LA = complete diet with palm kernel fatty acids rich in lauric acid; LBC-A42 = Lohmann-Brown-Classic, dissection after 42 days; LBC-A98 = Lohmann-Brown-Classic, dissection after 98 days.

*180 birds per diet allocated to 12 groups

^†^90 birds per age level allocated to 6 groups.

Lowercase letters (^a,b^) describe differences between groups depending on diet and age at *P*<0.05.

Between the breeds, there were no significant differences in the number of *C*. *jejuni* in the caecal content of the birds (Table 10). On average, 8.46±0.62 log_10_ CFU/g *C*. *jejuni* were present in the caecal content.

**Table 10:** Quantitative counts of *C jejuni* in caecal content samples [log_10_ CFU/g] of experimentally infected birds depending on genetic breed.

Time	Genetic breed*							
	R308		Hb757		LD		LBC-A42	
	Mean	SD	Mean	SD	Mean	SD	Mean	SD
Dissection^†^	8.44	0.72	8.43	0.68	8.40	0.51	8.57	0.46

R308 = Ross 308; Hb757 = Hubbard JA 757; LD = Lohmann Dual; LBC-A42 = Lohmann-Brown-Classic, dissection after 42 days.

*90 birds per genetic breed allocated to 6 groups.

^†^No significant differences between breeds depending on time at *P*<0.05.

## Discussion

Bacteria of the genus *Campylobacter* spp. are the most common causes of bacterially induced diarrhoea in humans [31, 32]. For decades, poultry meat has been assumed to be the cause of the infection behind *Campylobacter* enteritis in humans [33, 34]. Currently, poultry products are still the most common source of infection for humans [34–36]. Intestinal colonisation of chickens results in faecal contamination of the carcasses during the slaughtering process [37, 38]. Therefore, a reduction in the prevalence and the level of excretion of *C*. *jejuni* is strived for at production level.

### Performance parameters

Offering a lauric acid rich diet led to significant lower ADWGs in chickens of the LBC-A98 breed (Table 4). In a previous study, a significantly concentration-dependent reduced feed intake with the use of propionic, caprylic, caprine and lauric acids starting from a concentration of 30 g/kg diet was seen [39]. As a control, maize seed oil was used [39]. Thereby, lauric acid showed the greatest negative effect on ADFI (up to d 29: 5.9 g lower feed intake per g/kg lauric acid added to the diet) presuming the taste or the hormonal regulation of intestinal motility and appetite to be responsible for these observations [39]. Consistent with reduced feed intake, growth was lower with higher concentrations [39]. In the present study, the lauric acid rich diet had no effect of on the FCR. The lauric acid concentration in the present study might be too low for the diet to have any effect on performance parameters in general. For older birds (LBC-A98) taste sensitivity might be higher and therefore the feed intake decreases.

Birds of the LBC line showed significant differences in performance parameters depending on age (Table 5). The body weight, the daily weight gains as well as the FCR were always significantly higher in the older birds. This observation can be explained by the natural, genetically determined growth curve under optimal husbandry conditions. The comparison of performance parameters depending on the genetic breed showed also significantly differences between all genetics. The R308 birds had always the highest body weight, the highest daily weight gains and the lowest FCR. The genetics HB757, LD and LBC-A42 followed in descending order. These observations can also be explained by the natural, genetically determined growth curve under optimal husbandry conditions.

### Lauric acid concentrations in the gastrointestinal tract

Using a lauric acid rich complete diet with higher levels of palm kernel fats as opposed to administrating a control diet resulted in significantly higher lauric acid concentrations at all analysed sites (crop, gizzard, anterior small intestine, posterior small intestine, caecum; Fig 2) in the gastrointestinal contents of broilers. The lauric acid concentration was significantly higher in the proximal areas than in the distal areas. Nevertheless, the average lauric acid concentrations (0.11±0.04 g/kg) achieved in the caecum in the experimental group was the lowest compared to the concentrations in the more cranial parts of the gastrointestinal tract. Lauric acid is resorbed extremely well and incorporated into the muscles [24]. Nevertheless, the concentrations were higher than effective inhibitory concentrations *in vitro* [6]. Concentrations less than 0.10 mg/mL lauric acid were able to reduce the bacterial DNA synthesis of *C*. *jejuni* in *in vitro* studies [6]. The caecal and cloacal crypts are the localisation in the intestinal tract with the highest counts of *C*. *jejuni* along the gastrointestinal tract [40]. Therefore, an effect of lauric acid on Campylobacter is fundamentally possible when taken orally, especially during the passage on the way to the main colonisation site, the caecum.

### Effect of lauric acid supplementation on *Campylobacter* challenge

An effect of lauric acid on *C*. *jejuni* requires the presence of the acid in significant concentrations at the main site of colonisation, the caecum. In the present study the highest concentrations of lauric acid in the gastrointestinal tract were demonstrated in the cranial parts (Fig 2). A direct effect of the lauric acid in cranial areas is therefore only to be assumed in the initial infection. In the present investigations, in the initial phase the spread of the infection was delayed (Table 6). The proportion of cloacal swabs with positive *C*. *jejuni* detection was significantly lower in those birds receiving a lauric acid-rich complete diet. On its way to the actual colonisation site, *C*. *jejuni* comes into contact with very high lauric acid concentrations in the anterior regions of the gastrointestinal tract. Therefore, in a short time-frame of 24 hours until establishment of the infection [41], an effect might be possible. Under conditions of infection, *C*. *jejuni* recedes into the mucus layer of the crypts[40] and becomes difficult to reach for antimicrobial acting substances [42].

Although the concentrations of lauric acid in the caecal content are comparable to the inhibitory concentrations *in vitro* [6], it remains questionable whether these low concentrations are effective *in vivo*, too. We found no differences concerning *C*. *jejuni* counts in excreta samples of experimentally infected birds (Table 7) or in the intestinal content of the caecal content depending on diet (Table 9). *C*. *jejuni* is primarily found in the mucus layer of the caecal and cloacal crypts when colonising the gastrointestinal tract [40]. Some of the substances identified as effective against Campylobacter *in vitro* were less effective in *in vivo* studies [42–44]. Initially, (experimental infection with *C*. *jejuni* VFU612, 10^6^CFU/bird) caprylic acid had a significant effect on the diet of broilers (2.5 and 5 g/kg of diet) on reducing *C*. *jejuni* excretion (up to 1.8 log10 CFU/g; [45]). However, as in our study, the dietary concept did not lead to differences in caecal content on the day of dissection (d42).

Palm kernel products also contain caprylic and capric acid. However, the concentrations used in our diets were very low (0.12% based on dry matter; Table 3). These concentrations are rather low compared to the literature. Supplementing the diet with 0.35% and 0.70% caprylic acid consistently decreased (P<0.05) the colonisation of *C*. *jejuni* in the chicken caeca compared with positive control treatment [46].

To conclude, offering a lauric acid rich diet only led to low concentrations of the acid in the substantial section caecum and cloaca. Therefore, downstream effects of resorption of the acid and therefore higher lauric acid concentrations in muscles might be of greater importance. Campylobacter load in breast meat of Ross 308 broilers receiving a lauric acid rich diet was reduced during six days of storage (4°C) from initially 5.9 log_10_ CFU/g to 3.5 log_10_ CFU/g (treatment) compared to 4.3 log_10_ CFU/g (control; P = 0.0295; [24]).

### Campylobacter–age effects

From the results of the present study, it is apparent that the age of birds at the time of infection has a great influence on the frequency of the excretion of *C*. *jejuni* (Table 6). In older chickens of the laying breed (LBC-A98) the spreading of the colonisation was faster as was its disappearance. The *C*. *jejuni* numbers in excreta of LBC-A90 chickens ($\bar{x}$ log_10_ = 5,20±0,93 CFU/g) was also in the upper range of the *C*. *jejuni* numbers in broilers which are described as log 2–6 CFU/g excreta in the literature [40, 47]. Glünder [48] already showed that ten-week-old birds compared to four- and seven-week-old birds had a lower excretion. These observations coincide with our own results (Table 7). Young starlings were more frequently infected with *C*. *jejuni* compared to adult ones [49]. According to some authors, the probability of *Campylobacter* colonisation also increases with age [50–53]. However, the excretion is sometimes intermittent [54], but, on the other hand, numerically lower. This agrees with the investigations of Glünder [48] and our own investigations.

The age of birds at the time point of challenge had an influence on the detection of *C*. *jejuni* in the caecum 21 days after experimental challenge in our seeder model (Table 9). The *C*. *jejuni* counts in the caecum of the LBC-A98 breed were significantly lower (ΔCFU/g = log_10_1.91) than the *C*. *jejuni* number in the caecum of LBC-A42 chickens. The *C*. *jejuni* counts in the caecum of LBC-A42 chickens averaged at log_10_8.46±0.62 CFU/g caecal content. These concentrations are in the upper range of the *C*. *jejuni* numbers described in the literature in the caecum of log_10_ = 5–8 CFU/g caecal content [37, 40, 55, 56]. The reason for the lower detection rates in certain age groups is discussed; the composition of caecal flora, the developmental status of the immune system as well as the maturity of the immune system being responsible for this [57]. Also, use of these protective bacteria in poultry production can greatly reduce carriage of *C*. *jejuni* within poultry [58]. This approach may be important in the present study. *Campylobacter* spp. is predominantly found in the mucus layer that lies within the crypts [40]. Campylobacter is chemotactically attracted by L-fucose in the mucin, using mucin itself as a substrate for growth [59] and replicates in the mucus [60]. It is known from previous studies that bacteria that have similar properties produce metabolites antagonistic to *C*. *jejuni* [58]. Occupying the same niche in chicks can prevent colonization of most chicks by *C*. *jejuni* [58]. In that study, hens with cloacal samples negative for C. *jejuni* were screened for isolates expressing inhibitory metabolites to *C*. *jejuni* and simultaneously use mucin as a sole substrate for their growth [58]. The older birds in the present study might also have been the host of a more competent intestinal flora. The relative mucin availability increases with age. Therefore, perhaps the older birds (LBC-A98) already had a flora that had long been adapted to the degradation of larger amounts of mucin. Thus, there may have been some competition between the resident microflora and *C*. *jejuni* after experimental infection which led to a lower prevalence and lower counts in the caecal content.

### Campylobacter–effects of genetic breed

In the present investigations, an influence of the genetics on the *Campylobacter* prevalence (Table 6), but even more clearly on the amount of the excretion of *Campylobacter* with the excreta could be demonstrated (Table 8). The mean logarithmic *Campylobacter* numbers in the excreta of the LD birds and of the laying hybrids LBC-A42 showed significant differences to the two broiler breeds R308 and Hb757 at the end of the experimental period. For LBC-A42, these differences amounted to almost 2 log steps (ΔCFU/g = log 1.85) compared to the Hb757 breed. However, the LD breed showed the lowest Campylobacter prevalence 21 days after experimental challenge. From the literature, a link between genetic breed and *Campylobacter* prevalence in the group and bacterial counts of *C*. *jejuni* in the caecum is demonstrated [61–63]. Modern, fast-growing breeds responded with a strong inflammatory response to *C*. *jejuni* infections [19]. This was associated with a negative effect on the function of the T-lymphocytes, which led to a reduced immune regulation and damage to the intestinal mucosa with subsequent diarrhoea [19]. This relationship is consistent with our findings. On the other hand, when comparing a broiler and a laying genetic (Ross 308 versus Lohmann Selected Leghorn), 14 days after experimental infection (day one of life), there were significantly higher counts of *C*. *jejuni* in the caecum of the laying breed [64]. However, the sample size (n = 6 per group) was very small [64]. Even if the age is identical, it may be necessary to consider indirect effects of breed. For example, mucin concentrations also depend on the relative levels of protein uptake [65], and thus may have an effect on the *C*. *jejuni* infection dynamics.

## Conclusions

In summary, it can be said that so far no comparable studies have been conducted that have tested, on the one hand, possible effects of lauric acid on the outcome of an experimental *C*. *jejuni* challenge *in vivo*, and on the other hand, effects of age for different genetic breeds under standardised conditions. The effects of the dietary concept were successful in the initial phase of the infection. This may be relevant for the practice of late infections in flocks, for example, due to pre-catch, as common found in Germany.

The favourable effect of age was evident. This is in fact positive for alternative livestock systems which work with longer fattening periods. However, this only works if at the same time an entry of *C*. *jejuni* from outside can be efficiently prevented in these systems.

Various risk assessment models for evaluating *C*. *jejuni* have shown that for differences of 2 log steps in *C*. *jejuni* counts in the intestine of broilers, the incidence of human campylobacteriosis is decreased by 76%–98% [66] or 44%–95% [67]. A reduction in the prevalence by a factor of two, results in a two-fold reduction of human campylobacteriosis cases since a one-to-one ratio prevails between these two parameters [68]. Thus, any reduction in the primary production level with respect to the extent of the excretion (*Campylobacter* numbers) and the excretion rate (*Campylobacter* prevalence) is to be considered successful since the risk of human campylobacteriosis is significantly reduced.

Further studies with experimental challenge are necessary with the described infection model. The timespan between challenge and dissection should be shorter and possible effects of a late infection (due to pre-catch) as well as a less intensive bird to bird contact as in this experimental set-up should be proved in practice.
